# Targeted Metabolomics Highlights Dramatic Antioxidant Depletion, Increased Oxidative/Nitrosative Stress and Altered Purine and Pyrimidine Concentrations in Serum of Primary Myelofibrosis Patients

**DOI:** 10.3390/antiox13040490

**Published:** 2024-04-19

**Authors:** Renata Mangione, Cesarina Giallongo, Andrea Duminuco, Enrico La Spina, Lucia Longhitano, Sebastiano Giallongo, Daniele Tibullo, Giuseppe Lazzarino, Miriam Wissam Saab, Arianna Sbriglione, Giuseppe A. Palumbo, Andrea Graziani, Amer M. Alanazi, Valentina Di Pietro, Barbara Tavazzi, Angela Maria Amorini, Giacomo Lazzarino

**Affiliations:** 1 Department of Basic Biotechnological Sciences, Intensive and Perioperative Clinics, Catholic University of the Sacred Heart of Rome, Largo F. Vito 1, 00168 Rome, Italy; renata.mangione@unicamillus.org; 2 Departmental Faculty of Medicine, UniCamillus—Saint Camillus International University of Health and Medical Sciences, Via di Sant’Alessandro 8, 00131 Rome, Italy; andrea.graziani@unicamillus.org (A.G.); giacomo.lazzarino@unicamillus.org (G.L.); 3 Department of Medical and Surgical Sciences and Advanced Technologies “G.F. Ingrassia”, Division of Hematology, University of Catania, Via S. Sofia 87, 95123 Catania, Italy; cesarina.giallongo@unict.it (C.G.); sebastiano.giall@gmail.com (S.G.); palumbo.ga@gmail.com (G.A.P.); 4 Hematology Unit with BMT, A.O.U. Policlinico “G.Rodolico-San Marco”, Via S. Sofia 78, 95123 Catania, Italy; andrea.duminuco@gmail.com; 5 Department of Biomedical and Biotechnological Sciences, Division of Medical Biochemistry, University of Catania, Via S. Sofia 97, 95123 Catania, Italy; enricolaspina@outlook.it (E.L.S.); lucia.longhitano@unict.it (L.L.); d.tibullo@unict.it (D.T.); lazzarig@unict.it (G.L.); mirisaab@gmail.com (M.W.S.); arianna9820@gmail.com (A.S.); 6 Pharmaceutical Biotechnology Laboratory, Department of Pharmaceutical Chemistry, College of Pharmacy, King Saud University, Riyadh 11451, Saudi Arabia; amalanazi@ksu.edu.sa; 7 Neurotrauma and Ophthalmology Research Group, School of Clinical and Experimental Medicine, College of Medical and Dental Sciences, University of Birmingham, Edgbaston, Birmingham B15 2TT, UK; v.dipietro@bham.ac.uk; 8 National Institute for Health Research Surgical Reconstruction and Microbiology Research Centre, Queen Elizabeth Hospital, Edgbaston, Birmingham B15 2TH, UK

**Keywords:** antioxidants, ascorbic acid, GSH, HPLC, oxidative/nitrosative stress, primary myelofibrosis, purines, pyrimidines, serum

## Abstract

To date, little is known concerning the circulating levels of biochemically relevant metabolites (antioxidants, oxidative/nitrosative stress biomarkers, purines, and pyrimidines) in patients with primary myelofibrosis (PMF), a rare form of myeloproliferative tumor causing a dramatic decrease in erythropoiesis and angiogenesis. In this study, using a targeted metabolomic approach, serum samples of 22 PMF patients and of 22 control healthy donors were analyzed to quantify the circulating concentrations of hypoxanthine, xanthine, uric acid (as representative purines), uracil, β-pseudouridine, uridine (as representative pyrimidines), reduced glutathione (GSH), ascorbic acid (as two of the main water-soluble antioxidants), malondialdehyde, nitrite, nitrate (as oxidative/nitrosative stress biomarkers) and creatinine, using well-established HPLC method for their determination. Results showed that PMF patients have dramatic depletions of both ascorbic acid and GSH (37.3- and 3.81-times lower circulating concentrations, respectively, than those recorded in healthy controls, *p* < 0.0001), accompanied by significant increases in malondialdehyde (MDA) and nitrite + nitrate (4.73- and 1.66-times higher circulating concentrations, respectively, than those recorded in healthy controls, *p* < 0.0001). Additionally, PMF patients have remarkable alterations of circulating purines, pyrimidines, and creatinine, suggesting potential mitochondrial dysfunctions causing energy metabolism imbalance and consequent increases in these cell energy-related compounds. Overall, these results, besides evidencing previously unknown serum metabolic alterations in PMF patients, suggest that the determination of serum levels of the aforementioned compounds may be useful to evaluate PMF patients on hospital admission for adjunctive therapies aimed at recovering their correct antioxidant status, as well as to monitor patients’ status and potential pharmacological treatments.

## 1. Introduction

Primary myelofibrosis (PMF) is a rare form of myeloproliferative tumor (prevalence 1–9:100,000) in which clonal hyperproliferation of stem cell-derived mature myeloid lineages (erythrocytes, leukocytes, megakaryocytes) are associated with bone marrow fibrosis (caused by abnormal collagen and/or reticulin production) causing a dramatic decrease in erythropoiesis and angiogenesis [1]. Furthermore, osteosclerosis, atypic megakaryocytes, extramedullary hematopoiesis, and abnormal cytokine expression are additional characteristics of PMF patients [2]. Following diagnosis, the median overall survival of PMF patients is 6–7 years, and the most common causes of death include leukemic transformation, vascular events, and infections [3,4].

Systemic inflammation plays a central role in the pathogenesis and progression of myelofibrosis due to hyperactivated signaling pathways leading to the overproduction of inflammatory cytokines [5,6]. Moreover, in recent years, oxidative stress has been demonstrated to have a crucial role in both the pathogenesis of PMF and its transformation into acute myeloid leukemia (AML) [7,8]. Indeed, chronic sustained inflammation might induce a state of chronic oxidative stress in the bone marrow (BM), thereby inducing mutations in hematopoietic stem cells (HSCs) [2,9]. When this genetic insult occurs, the clone itself constantly produces inflammatory factors (such as TGF-β and possibly other cytokines produced as a result of hyperactivated JAK2 kinase), creating a vicious circle that predisposes a high-risk BM microenvironment [7,10]. Furthermore, the stromal change occurring in PMF patients is secondary to the cytokine “storm” secreted by the hematopoietic clone [11].

Chronic inflammation is characterized by constant activation of immune cells, DNA damage, genomic instability, tissue destruction, remodeling, and fibrosis, which are all features of myelofibrosis [12]. Of note, chronic inflammation is also associated with aberrant DNA methylation, which is increased in PMF patients [13,14,15]. Both clonal myeloproliferation per se and inflammatory factors cause in vivo leukocytes, platelets, and endothelial activation, which sustain, in turn, oxidative stress conditions in the bloodstream, thus increasing the risk of thrombosis in these patients [16]. Compared to their healthy counterpart, MF CD34+ cells are characterized by sustained oxidative stress conditions, a decrease in superoxide dismutase activity, and an increase in DNA damage [17]. Moreover, MF CD34+ cells harboring calreticulin (CALR) mutation exhibit higher levels of ROS and apoptosis than those with the Janus kinase (JAK) 2617F mutation [17]. JAK inhibitors act by reducing the activity of the JAK-STAT pathway, and the newly approved molecules also evidenced a reduction in the grade of BM fibrosis [18,19].

However, no studies have been performed to evaluate the levels of circulating antioxidants, which could be crucial to counteract the long-term impact of a sustained state of oxidative stress in PMF patients. Additionally, a full evaluation of the purine and pyrimidine profiles in the serum of PMF patients has not yet been reported, although recent studies demonstrated that serum uric acid concentrations in these subjects are significantly higher than those found in controls [20] and are often associated with worse patients’ outcome [21]. Therefore, the measurement of serum uric acid has been suggested as a valuable prognostic indicator to be used in PMF patients. However, these studies did not point out whether the increase in uric acid was connected to a more generalized dysmetabolism of purine compounds, particularly those connected to uric acid formation (hypoxanthine and xanthine). Furthermore, given the antioxidant properties attributed to uric acid, it has not been indicated whether these hyperuricemic PMF patients had lower signs of ROS-mediated oxidative stress.

In previous studies, we had the opportunity to perform targeted metabolomic evaluations of serum/plasma samples of patients suffering from different pathological conditions [22,23]. Invariably, a decrease in antioxidants, an increase in purine and pyrimidine compounds, and an increase in oxidative/nitrosative stress biomarkers characterized the different biological specimens obtained from the aforementioned cohorts of patients compared to the values measured in matched control groups. Moreover, changes in the circulating levels of several of these compounds correlated with the disease progression and with the patient’s clinical conditions [24]. Herein, we undertook the present study to quantitatively measure water-soluble antioxidants, purines, pyrimidines, and oxidative/nitrosative stress biomarkers in serum samples of PMF patients with the aim of finding correlations with parameters reflecting their clinical conditions and possibly highlighting new biochemical dysfunctions characterizing PMF patients.

## 2. Materials and Methods

### 2.1. Patients’ Recruitment and Criteria for Inclusion in the Study

The study was approved by the Ethics Committee of the Azienda Ospedaliero-Universitaria Policlinico “G.Rodolico-San Marco” (protocol number 54/2022/PO 743), and written informed consent was obtained from all patients, according to the Declaration of Helsinki. Peripheral blood samples were obtained from thirty non-consecutive patients with a suspected diagnosis of PMF. Assessment of PMF was confirmed in twenty-two of them, according to the recently updated International Consensus Classification (ICC) criteria [25]. Only those patients naïve to pharmacological treatments for PMF were included in this study. Exclusion criteria were renal failure (acute or chronic), prior hematopoietic bone marrow transplant, and diagnosis of myelofibrosis secondary to essential thrombocythemia or polycythemia vera. A group of 22 age-matched healthy volunteers was used as the control group. Healthy controls who suffered from any inflammatory pathology, allergic manifestation, bacterial and/or viral infection in the previous 30 days or who used nutraceutical/adjuvant supplementation were not included in the control group.

### 2.2. Blood Sampling and Serum Processing for the HPLC Analysis of Metabolites

Peripheral blood samples were collected from the antecubital vein into a single VACUETTE polypropylene tube containing a serum separator and clot activator (Greiner-Bio One GmbH, Kremsmunster, Austria). Fasting PMF patients and controls were allowed to rest for at least 15 min before carrying out blood withdrawal, with the procedure performed between 8.00 and 9.00 a.m. To separate serum, blood withdrawals were kept for 30 min at room temperature and then centrifuged at 1890× *g* for 10 min. Serum samples were transferred into a new tube, and an aliquot of 500 μL was used for the subsequent organic solvent deproteinization [26]. Briefly, proteins were removed by the addition of 1 mL of ice-cold far UV, HPLC-grade acetonitrile to 0.5 mL of serum. After vigorous vortexing for 90 s, samples were centrifuged at 20,890× *g* for 10 min at 4 °C, supernatants were collected and transferred to a new tube, supplemented with 3 mL chloroform, vigorously vortexed for 120 s, and again centrifuged at 20,890× *g* for 10 min at 4 °C. The upper aqueous phase was collected and again extracted with chloroform to remove the organic solvent (acetonitrile). The resulting aqueous phase, free of proteins, was ready for the high-performance liquid chromatographic (HPLC) analyses of hydrophilic, low-molecular-weight metabolites.

### 2.3. Analysis of Purines, Pyrimidines, Antioxidants, and Oxidative/Nitrosative Stress Biomarkers by HPLC

The high-performance liquid chromatographic (HPLC) separation of hypoxanthine, xanthine, uric acid (as representative purines), uracil, β-pseudouridine, uridine (as representative pyrimidines), reduced glutathione (GSH), ascorbic acid (as two of the main water-soluble antioxidants), malondialdehyde, nitrite, nitrate (as oxidative/nitrosative stress biomarkers) and creatinine (indirectly connected to energy metabolism) was performed as previously described [23,24,26]. A Surveyor HPLC system, equipped with a highly sensitive photodiode array detector, supporting a 5-cm light-path flow cell and set up between 200 and 300 nm wavelengths for signal acquisition, was used (Thermo Fisher Scientific Italia, Rodano, Milan, Italy). Chromatographic acquisition and post-processing analysis were performed by a PC using the ChromQuest^®^ software package, 5.0 version, provided by the HPLC manufacturer. To obtain the separation of the compounds of interest, a Hypersil 250 × 4.6 mm, 5 µm particle-size column, provided with its own guard column (Thermo Fisher Scientific Italia, Rodano, Milan, Italy), was utilized.

The isocratic, ion-pairing separation of the compounds under evaluation was obtained using a mobile phase containing 12 mM tetrabutylammonium hydroxide (as the pairing reagent), 10 mM KH_2_PO_4_, 0.125% methanol, pH 7.00. A flow rate of 1.2 mL/min and a column temperature of 10 °C were maintained constant throughout the analysis. Assignment of peaks in chromatographic runs of protein-free serum samples was performed by comparing retention times and absorption spectra of peaks in chromatographic runs of freshly prepared ultrapure standards. The concentrations of the different compounds in serum extracts were determined at the wavelengths of 206 (GSH, nitrite, and nitrate), 234 (creatinine), and 260 nm (purines, pyrimidines, and ascorbic acid), comparing the areas of the peaks of the compounds of interest with those obtained in chromatographic runs of standard mixtures with known concentrations.

### 2.4. Statistical Analysis

Statistical analysis was performed using the GraphPad Prism program, release 8.01 (GraphPad Software, San Diego, CA, USA). The Kolmogorov-Smirnov test was applied to determine the normal distribution of the data. The median age of the PMF patients and control groups was considered as the threshold value to divide them into two subgroups of younger and older subjects. Mann-Whitney test was used to compare ranks between serum compounds and patients’ demographic data or myelofibrosis characteristics. Differences between the two groups for each compound considered were determined using the two-tailed Student’s *t*-test for unpaired samples. Values of *p* < 0.05 were regarded as statistically significant.

## 3. Results

### 3.1. Clinical Features of PMF Patients

Table 1 summarizes the demographic data and clinical features of the PMF patients (*n* = 22) and healthy controls (*n* = 22) included in the study. A prevalence of males occurred in this cohort of PMF patients, with females being half the number of males. Blood counts had a large range of variability. Patients with blast accounted for only 5% of the total. Most of them were classified into 0, and 1 BM fibrosis, and 14/22 carried the JAK2 as the driver mutation. Based on risk class, those with an IPSS intermediate-2 value were the most abundant.

### 3.2. PMF Patients Have Dramatic Depletion of Serum Ascorbic Acid and Concomitant Marked Biochemical Evidence of Sustained Oxidative/Nitrosative Stress

**Figure 1:**
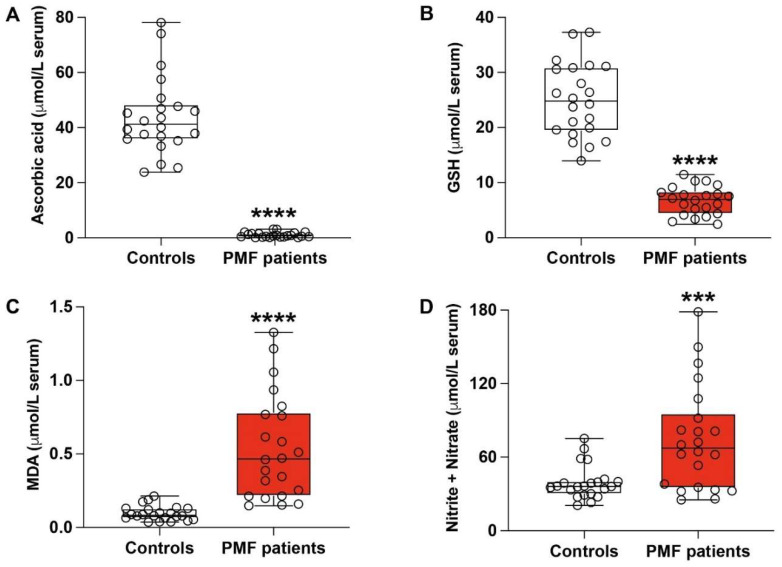
Concentrations of ascorbic acid (**A**), GSH (**B**), malondialdehyde (**C**), and nitrite + nitrate (**D**) were recorded by HPLC in serum samples of healthy controls and PMF patients. GSH = reduced glutathione; MDA = malondialdehyde. Box plots reporting all data points, minimum, median, maximum, 25, and 75 percentiles are shown. Significantly different from controls, *** *p* < 0.003; **** *p* < 0.0001.

Our data showed a dramatic decrease of ascorbic acid (Figure 1A) and GSH (Figure 1B) in serum samples of PMF patients, which was accompanied by relevant increases of malondialdehyde (MDA), an indicator of ROS-mediated lipid peroxidation (Figure 1C), and nitrite + nitrate (Figure 1D), as stable end-products of nitric oxide metabolism usable to evaluate nitrosative stress.

As far as ascorbic acid is concerned, its mean circulating levels in PMF patients were 1.13 ± 0.93 μmol/L serum, with a 37.3-fold decrease (*p* < 0.0001) compared to the mean values recorded in control healthy volunteers (42.19 ± 13.05 μmol/L serum). Concomitantly, the mean GSH in serum of PMF patients was 6.68 ± 2.64 μmol/L serum, showing a 3.81-fold decrease (*p* < 0.0001) in comparison with the mean values measured in controls (25.48 ± 6.78 μmol/L serum). This dramatic depletion of the principal water-soluble antioxidants was mirrored by the significant increase of serum MDA and nitrite + nitrate, strongly indicating a condition of sustained oxidative/nitrosative stress characterizing PMF patients. In particular, mean levels of serum MDA in PMF patients (0.52 ± 0.34 μmol/L serum) increased by 4.73-fold (*p* < 0.0001) in comparison with the mean values measured in controls (0.11 ± 0.06 μmol/L serum). Evidence of nitrosative stress in PMF patients was indicated by the 1.66-fold increase of the nitrite +nitrate serum concentrations compared to the values found in controls (*p* < 0.003). (*p* < 0.003).

### 3.3. PMF Patients Have Altered Serum Profile of Purines, Pyrimidines and Creatinine

**Figure 2:**
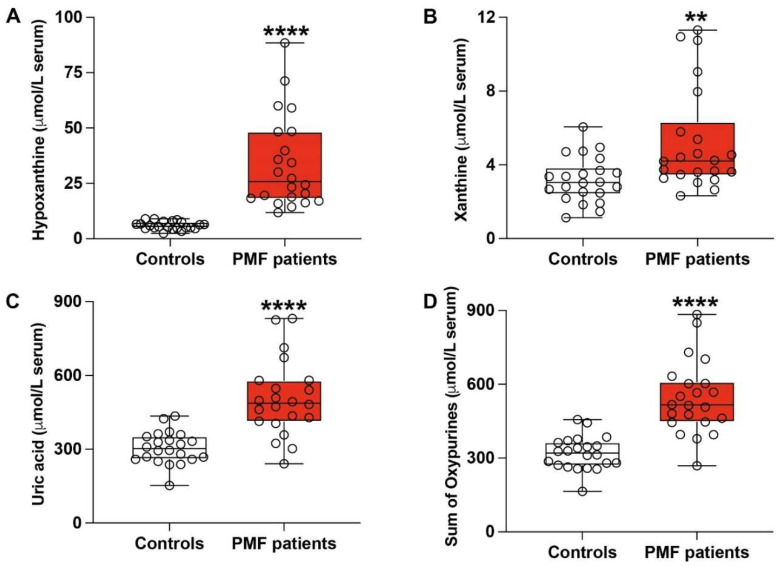
Concentrations of hypoxanthine (**A**), xanthine (**B**), uric acid (**C**), and sum of oxypurines (**D**) were recorded by HPLC in serum samples of healthy controls and PMF patients. In (**D**), the sum of oxypurines = hypoxanthine + xanthine + uric acid. Box plots reporting all data points, minimum, median, maximum, 25, and 75 percentiles are shown. Significantly different from controls, ** *p* < 0.01, **** *p* < 0.0001.

Figure 2 illustrates the circulating concentrations of purines (hypoxanthine, xanthine, and uric acid) recorded in the groups of healthy controls (*n* = 22) and PMF patients (*n* = 22).

Circulating concentrations of hypoxanthine in the serum of PMF patients were three times higher than those measured in controls (33.77 ± 20.77 μmol/L vs. 5.97 ± 1.81 μmol/L; *p* < 0.0001; Figure 2A). Similarly (Figure 2B,C), both serum levels of xanthine (5.26 ± 2.82 μmol/L) and uric acid (505.12 ± 153.34 μmol/L) were significantly increased in PMF patients compared to the corresponding values found in healthy donors (3.19 ± 1.22 μmol/L and 306.08 ± 65.19 μmol/L, respectively; *p* < 0.01 and *p* < 0.0001). Consequently (Figure 2D), the sum of circulating oxypurines (hypoxanthine + xanthine + uric acid), originating from the degradation pathway of purine nucleotides, was significantly higher in PMF patients (544.26 ± 149.64 μmol/L) than in controls (319.11 ± 66.96 μmol/L; *p* < 0.0001).

**Figure 3:**
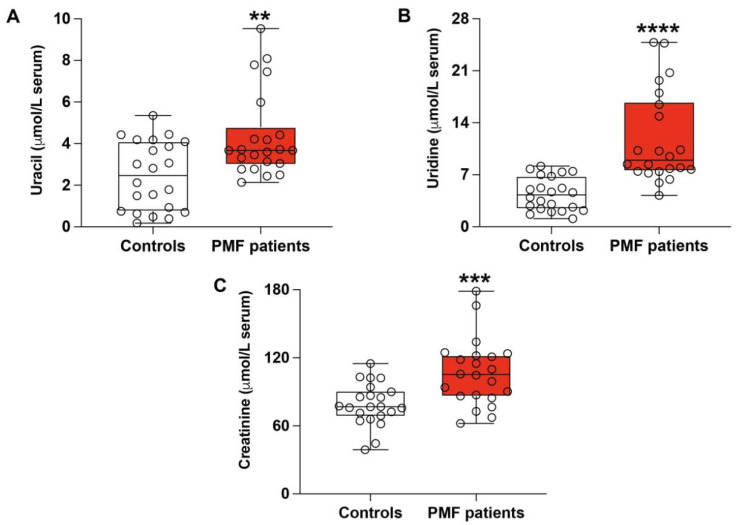
Concentrations of uracil (**A**), uridine (**B**), and creatinine (**C**) were recorded by HPLC in serum samples of healthy controls and PMF patients. Box plots reporting all data points, minimum, median, maximum, 25, and 75 percentiles are shown. Significantly different from controls, ** *p* < 0.01, *** *p* < 0.0001, **** *p* < 0.0001.

Indications of metabolic imbalance occurring in PMF patients were also confirmed by the concentrations of pyrimidines and creatinine in their serum samples. Indeed, (Figure 3A,B), the amounts of uracil and uridine in the serum of PMF patients were, respectively, 4.35 ± 2.06 and 11.75 ± 6.22 μmol/L, whilst those measured in controls were, respectively, 2.46 ± 1.64 and 4.39 ± 2.24 μmol/L (*p* < 0.01 and *p* < 0.0001). Moreover (Figure 3C), PMF patients also showed increased creatinine levels (from 78.84 ± 18.46 μmol/L observed in serum of healthy controls to 106.5 ± 29.35 μmol/L; *p* < 0.003).

### 3.4. ROC Curves of Specific Metabolites Allow Clustering PMF Patients and Controls into Two Distinct Groups on the Basis of Their Respective Serum Metabolic Profiles

**Figure 4:**
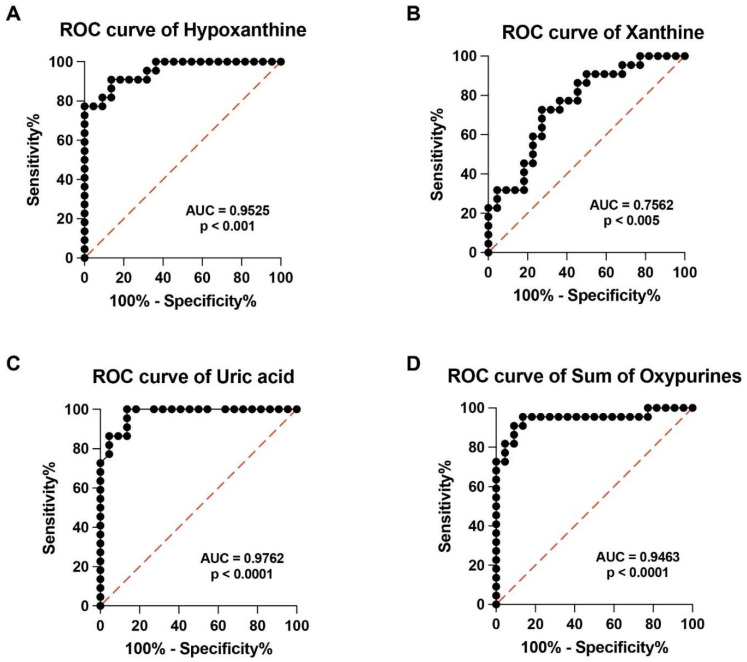
Receiver Operating Characteristic (ROC) curves of the serum values of hypoxanthine (**A**), xanthine (**B**), uric acid (**C**), and sum of oxypurines (**D**) of the two cohorts of control healthy volunteers and PMF patients. The Area Under the Curve (AUC) and significances are reported for each ROC curve. The sum of oxypurines = hypoxanthine + xanthine + uric acid.

**Figure 5:**
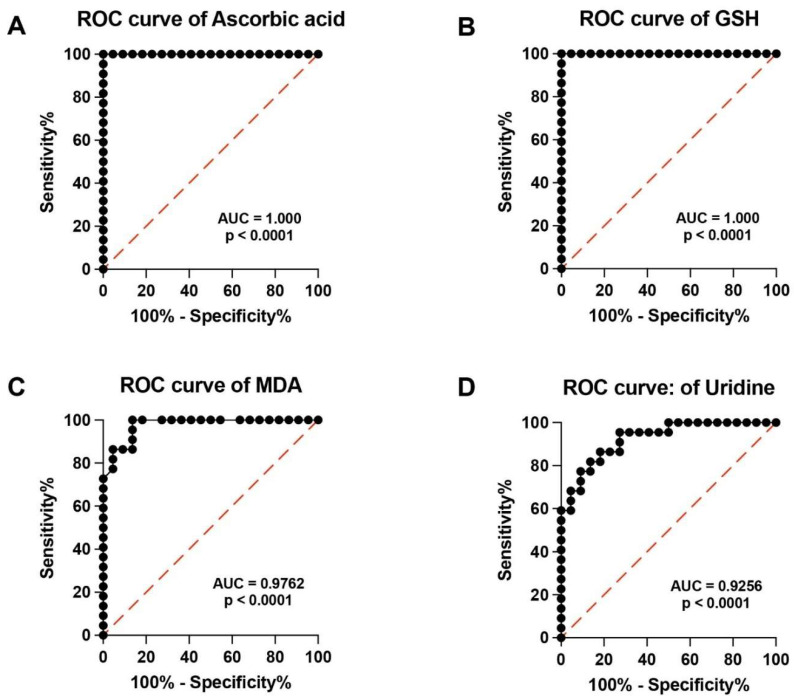
Receiver Operating Characteristic (ROC) curves of the serum values of ascorbic acid (**A**), GSH (**B**), MDA (**C**), and uridine (**D**) of the two cohorts of control healthy volunteers and PMF patients. The Area Under the Curve (AUC) and significances are reported for each ROC curve. GSH = reduced glutathione; MDA = malondialdehyde.

In order to evaluate the potential clinical impact of performing a targeted metabolomic evaluation of serum samples of PMF patients, we calculated the Receiver Operating Characteristic (ROC) curves of analyzed metabolites. High specificity and sensitivity were found in ROC curves of hypoxanthine, xanthine, uric acid, sum of oxypurines, (Figure 4A–D), ascorbic acid, GSH, MDA and uridine (Figure 5A–C).

Although all the AUCs indicated in the Figures are indicative of high sensitivity and specificity, those of ascorbic acid and GSH strongly suggest that the determination of these compounds in serum may be highly useful in the laboratory hematological evaluation of PMF patients.

### 3.5. Correlation between Targeted Metabolomics and PMF Characteristics

In order to find potential correlations between each serum metabolite analyzed, and any demographic or clinical feature, our cohort of PMF patients was divided into different subgroups, according to the data shown in Table 1. In particular, PMF patients’ subgroups of younger (<68 years of age, *n* = 11) and older (>68 years of age, *n* = 11), males (*n* = 15) and females (*n* = 7), low BM fibrosis (0–1, *n* = 13) and high BM fibrosis (2–3, *n* = 9), as well as low IPSS (low-int-1 risk class, *n* = 7) and high IPSS (int-2-high risk class, *n* = 15), were obtained. Serum levels of metabolites of each of the aforementioned subgroups were compared using the two-tailed Student’s *t*-test for unpaired samples.

As shown in Table 2, where numbers in bold indicate significant *p* values, we found that younger PMF patients had lower serum hypoxanthine (19.63 ± 8.52 μmol/L) compared to the value recorded in older PMF patients (44.31 ± 28.26 μmol/L, *p* < 0.024). As expected, higher values of serum creatinine were detected in males (118.87 ± 42.65 μmol/L) compared to the concentrations measured in females (80.55 ± 33.39 μmol/L, *p* < 0.004).

Concerning the subgroups with different clinical characteristics, we found that serum uridine concentrations and grade of BM fibrosis (13.63 ± 6.72 and 7.45 ± 2.12 μmol/L for lower and higher grade, respectively, *p* < 0.03), and uracil with IPSS risk class (5.22 ± 1.84 and 3.92 ± 2.17 μmol/L for lower IPSS and higher, respectively, *p* < 0.04). Moreover, we also observed a significant correlation between the sum of nitrates with the IPSS risk class (104.88 ± 44.43 and 56.99 ± 33.14 μmol/L for lower and higher IPSS, respectively, *p* < 0.02).

## 4. Discussion

In the present study, by measuring serum metabolites using a targeted metabolomics approach, allowed to evidence, for the first time, that PMF patients are characterized by circulating alterations of specific low-molecular weight metabolites, indicative of depleted antioxidant defenses with sustained oxidative/nitrosative stress, as well as of energy metabolism impairment potentially due to mitochondrial dysfunction.

Since PMF is a rare hematological disease, there is definitely a low number of clinical studies compared to other more common hematological diseases. Therefore, very little data can be obtained from the literature regarding the molecular mechanisms of onset and progression of the disease, which may have a significant influence on the circulating levels of different metabolites and may be helpful in the laboratory diagnosis and monitoring of PMF.

Among these mechanisms, previous studies demonstrated that PMF patients had lower values of serum native thiol, total thiol, disulfide levels, disulfide/native thiol, and disulfide/total thiol ratios compared to the values measured in healthy controls [27]. Djikic and colleagues also demonstrated lower serum antioxidant capacity in PMF patients associated with higher MDA and nitrite + nitrate concentrations [28]. According to the results of the present study, it is possible to affirm that the decreased serum antioxidant capacity in PMF patients is due to the dramatic depletions of their circulating ascorbic acid and GSH concentrations.

The relevant biological role of ascorbic acid in the insurgence and progression of cancer has been demonstrated by numerous studies, highlighting the multiple modes of action of ascorbic acid in tumors, including hematological malignancies [29,30]. These mechanisms involve the regulatory activity of ascorbic acid to (i) modulate the expression of Ten-Eleven Translocases DNA demethylases 2 (TET2), one of the principal epigenetic regulators of normal and malignant hematopoiesis [31,32]; (ii) decrease the synthesis of the carcinogen nitroso compound [33]; (iii) inhibit glycolysis through indirect inhibition of glyceraldehyde-3-phosphate dehydrogenase [34]; (iv) counteract the metastatic process by inhibiting expression and activity of hypoxia-inducible factor-1α (HIF-1α) [35]; (v) increase the activities of lysyl- and prolyl-hydroxylases, favoring collagen synthesis [36,37]. Our finding strongly supports the hypothesis that ascorbic acid depletion may represent, even in PMF, a crucial determinant involved in this cancer development. In our cohort of PMF patients, we did not find a significant correlation between concentrations of circulating ascorbic acid and BM fibrosis. It may be hypothesized that low serum levels of this metabolite are due to higher consumption rates of ascorbic acid caused by the increased collagen synthesis occurring in PMF.

Together with ascorbic acid depletion, our cohort of PMF patients was also characterized by a remarkable decrease in serum GSH, further compromising the circulating reservoirs of water-soluble antioxidants. This finding is in accordance with the aforementioned study showing that PMF patients had lower values of serum thiols [28,38]. Lower GSH availability may certainly impact even the serum levels of ascorbic acid. Indeed, cellular recycling of dehydroascorbic acid (the oxidized form of ascorbic acid) to ascorbic acid is strictly dependent on GSH concentrations. Decreasing the efficiency of this redox cycle because of low GSH levels may negatively affect cell ascorbic acid regeneration, contributing to serum ascorbic acid depletion.

Serum GSH depletion has recently been found in cancer patients suffering from uterine myoma and endometrial cancer [39], as well as in those affected by lung cancer. The simultaneous decrease of ascorbic acid and GSH in PMF patients may be even more important in light of their crucial role in regulating the immune response [40,41]. Concomitant depletion of both compounds may seriously compromise the immune system, thereby creating optimal conditions that favor cancer insurgence and progression. To support this hypothesis, it is worth underlining that PMF patients often develop symptoms of autoimmune disorders and dysregulated immune system phenomena [42,43]. In addition, infections are the main causes of unfavorable prognosis and represent the cause of death in 10% of PMF patients [44].

Compared to control healthy volunteers, PMF patients showed a significant increase in serum oxypurines (hypoxanthine, xanthine, and uric acid), pyrimidines (uracil and uridine), and creatinine. Under different pathological conditions characterized by mitochondrial dysfunction with energy metabolism impairment [45], the increase in the circulating concentrations of the aforementioned compounds was associated with the disease progression and the patient’s clinical conditions [22,23,24]. It is, therefore, conceivable that, even in PMF patients, these serum alterations are connected to the malfunctioning of mitochondria. The consequent energy crisis, due to decreased mitochondrial capacity, generates ATP and activates degradation pathways, causing increased formation of dephosphorylated purines [46,47,48], as well as those of uridine [49,50] and creatinine [51]. Indeed, malfunctioning of mitochondria is a common finding in various types of both liquid and solid cancers [52]. As far as uric acid is concerned, it may be hypothesized that its serum increase is a defense mechanism caused by the decrease in ascorbic acid and GSH. In fact, uric acid has well-known antioxidant properties [53,54] that may be useful to partially counteract the serum ascorbic acid and GSH depletions and increase PMF-associated oxidative/nitrosative stress. However, it is worth recalling that this effect may be exerted by uric acid only extracellularly since its intracellular concentrations are too low to account for significant antioxidant activity within the cellular environment [55,56].

Considering the relationship between serum compounds and myelofibrosis characteristics, JAK2 mutated PMFs (as mentioned, present in about 60% of cases) show a lower creatinine value and higher xanthine and MDA, evidencing a potential role of hyperactivated JAK-STAT pathway in the oxidative stress in these patients. Starting from this assumption, a further evaluation of JAK2 inhibitors treatment for reducing these values could be studied, trying to identify better patients that can experience an adequate early response to these molecules, such as is reported with clinical data in the RR6 prognostic model [57]. Finally, we interestingly report a correlation between uracil and more aggressive PMF based on the IPSS score, suggesting a potential role of this pyrimidine for individuating patients with a worse prognosis.

It should, however, be underlined that the significance of these results is limited by the restricted number of patients enrolled in the study. Indeed, the relatively low number of patients did not allow subgroups of PMF patients with higher numerosity, thereby hindering the possibility to highlight clear correlations between serum metabolites and different PMF patients’ clinical features.

## 5. Conclusions

Results reported in the present study, although obtained in a relatively small number of subjects, highlighted, for the first time to the best of our knowledge, that PMF patients have severe depletion of water-soluble antioxidants in serum that is accompanied by parameters representative of increased oxidative/nitrosative stress (MDA and nitrite + nitrate) and by indicators of mitochondrial dysfunction with energy penalty (hypoxanthine, xanthine, uric acid, uridine and creatinine). When hematological screenings are carried out to corroborate the clinical diagnosis of PMF, the analysis of these compounds in serum may be useful to support the results of the other conventional hematological indicators. Furthermore, the progression of the disease and the effectiveness of anti-PMF drugs may possibly be followed through the determination of these metabolites in serum from peripheral blood.

## Figures and Tables

**Table 1:** Clinical characteristics of PMF patients (*n* = 22) and healthy controls (*n* = 22) were included in the study. Assessment of PMF was carried out following recently updated International Consensus Classification (ICC) criteria.

	Patients	Healthy Controls
**Median Years of Age [Range]**	68 [41–76]	63 [31–75]
**Sex**	15 M (68%), 7 F (32%)	13 M (59%), 9 (41%)
**Blood count**		
˗ Hb, g/dL [range]	10.5 [6.3–17.2]	13.8 [11.9–15.6]
˗ WBC, ×10^4^/mmc [range]	9.8 [1.3–149.1]	6.1 [4.3–9.6]
˗ N, ×10^4^/mmc [range]	7.2 [1.3–127]	3.8 [2.4–5.6]
˗ L, ×10^4^/mmc [range]	1.7 [0.4–12.2]	3.2 [0.9–5.2]
˗ M, ×10^4^/mmc [range]	0.6 [0.2–1.6]	0.5 [0.2–1.4]
˗ Platelets, ×10^4^/mmc [range]	347 [70–1014]	239 [146–401]
**Blast > 5%**	2 (9.1%)	-
**BM fibrosis**		-
˗ 0	3 (13.6%)	
˗ 1	10 (45.5%)	
˗ 2	6 (27.3%)	
˗ 3	3 (13.6%)	
**Driver mutations**		-
˗ JAK2	14 (63.71%)	
˗ CALR	3 (13.6%)	
˗ MPL	0	
˗ Triple negative	3 (13.6%)	
˗ Not available	2 (9.1%)	
**IPSS/MYSEC-PM**		-
˗ Low	2 (9.1%)	
˗ Intermediate-1	5 (22.7%)	
˗ Intermediate-2	10 (45.5%)	
˗ High	5 (22.7%)	

Hb: hemoglobin; WBC = white blood cells; N = neutrophils; L = Lymphocytes; M = monocytes; IPSS/MYSEC-PM = International Prognostic Scoring System/MYSEC Prognostic Model Risk Calculator.

**Table 2:** Values of *p* were calculated when comparing levels of measured serum metabolites in PMF patients divided according to their demographic and clinical data. PMF patients were categorized intodifferent subgroups according to age (<68 years of age, *n* = 11; >68 years of age, *n* = 11), sex (males, *n* = 15; females, *n* = 7), BM fibrosis (0–1, *n* = 13; 2–3, *n* = 9), and IPSS risk class (low-int-1, *n* = 7; int-2-high, *n* = 15).

	Hyp	Xan	UA	Ura	Uri	Creat	AA	GSH	MDA	Nitrite + Nitrate
**Age**, <67 years (*n* = 11) vs. >68 years (*n* = 11)	**0.024**	0.654	0.47	0.81	0.705	0.863	0.282	0.31	0.467	0.557
**Sex**, M (*n* = 15) vs. F (*n* = 7)	0.235	>0.999	0.34	0.424	0.97	**0.004**	0.968	0.733	0.97	0.842
**BM fibrosis**, 0–1 (*n* = 13) vs. 2–3 (*n* = 9)	0.916	0.595	0.645	0.651	**0.029**	>0.999	0.86	0.704	0.185	0.743
**Risk class, IPSS** Low-Int-1 (*n* = 7) vs. Int-2-High (*n* = 15)	0.287	0.361	0.443	**0.037**	0.535	0.856	0.804	0.332	0.11	**0.016**

Numbers in bold indicate significant differences between the corresponding subgroups. Hyp = hypoxanthine; Xan = xanthine; UA = uric acid; Ura = uracil; Uri = uridine; Creat = creatinine; AA = ascorbic acid; GSH = reduced glutathione; MDA = malondialdehyde; nitrite + nitrate; IPSS: International Prognostic Scoring System. The first three digits of exact *p* values are indicated.

## Data Availability

The datasets used and/or analyzed in this study are reported within the manuscript and/or additional files and are available from the corresponding authors.
